# The First Complete Plastid Genome from Joinvilleaceae (*J*. *ascendens*; Poales) Shows Unique and Unpredicted Rearrangements

**DOI:** 10.1371/journal.pone.0163218

**Published:** 2016-09-22

**Authors:** William P. Wysocki, Sean V. Burke, Wesley D. Swingley, Melvin R. Duvall

**Affiliations:** 1 Center for Data Intensive Sciences, University of Chicago, 5454 South Shore Dr., Chicago, IL 60615, United States of America; 2 Northern Illinois University; 1425 W. Lincoln Hwy, DeKalb, IL 60115, United States of America; Academia Sinica, TAIWAN

## Abstract

Joinvilleaceae is a family of tropical grass-like monocots that comprises only the genus *Joinvillea*. Previous studies have placed Joinvilleaceae in close phylogenetic proximity to the well-studied grass family. A full plastome sequence was determined and characterized for *J*. *ascendens*. The plastome was sequenced with next generation methods, fully assembled de novo and annotated. The assembly revealed two novel inversions specific to the Joinvilleaceae lineage and at least one novel plastid inversion in the Joinvilleaceae-Poaceae lineage. Two previously documented inversions in the Joinvilleaceae-Poaceae lineage and one previously documented inversion in the Poaceae lineage were also verified. Inversion events were identified visually and verified computationally by simulation mutations. Additionally, the loss and subsequent degradation of the *accD* gene in order Poales was explored extensively in Poaceae and *J*. *ascendens*. The two novel inversions along with changes in gene composition between families better delimited lineages in the Poales. The presence of large inversions and subsequent reversals in this small family suggested a high potential for large-scale rearrangements to occur in plastid genomes.

## Introduction

Among monocot Poales, the grass family is most conspicuous ecologically and important agronomically. Close relatives to the grasses are found in two families, Joinvilleaceae and Ecdeiocoleaceae [1], which are not as well studied, but suggest biogeographical, morphological, and phylogenomic aspects that bear on the evolutionary origins of the large grass radiation [1, 2, 3]. The two genera of Ecdeiocoleaceae are narrowly endemic to a restricted region of sand plains in extreme western Australia. Joinvilleaceae, which is more accessible, was selected for this study because of its previously reported and detailed plastome molecular evolutionary history [4].

Joinvilleaceae includes the single genus *Joinvillea*, which originally contained six species [5], but The Plant List [6] now recognizes four species: *J*. *ascendens*, *J*. *borneensis*, *J*. *bryanii*, and *J*. *plicata*. These species are found in often inaccessible localities of Malaysia, western Indonesia, and the Philippines. *Joinvillea* is also sparsely distributed in the Hawaiian archipelago and other remote Pacific islands where they may be introduced [6, 7]. *Joinvillea* species are robust, grass-like herbs with strongly plicate leaves and flowers that are borne in large, multibranched terminal panicles. The small, perfect flowers themselves are distinguished from those of grasses in having a conspicuous perianth of six tepals and non-plumose stigmas, both of which suggest insect pollination, possibly by ants [1]. After fertilization the ovaries develop into fleshy drupes [8], again contrasting with the caryopsis typical of Poaceae.

Most angiosperm plastid chromosomes (plastomes) are structured with two single copy regions, referred to as the large and small single copy (LSC and SSC) regions, flanked by two inverted perfectly repeated regions (IR) [9]. Compared to grasses, the plastomes of *Joinvillea* spp. were suggested to have undergone major kilobase-sized rearrangements indicative of large scale inversion events, suggested by PCR analyses primed from alternative sites in the LSC region [4, 10, 11]. Small inversions are often associated with stem-loop structures suggesting recombination in the stem-forming region as the causal mechanism [12]. However, larger inversions, like those in graminid Poales (Poaceae, Joinvilleaceae, Ecdeiocoleaceae, and Flagellariaceae), have been proposed to be the result of recombination. Hiratsuka et al. [13] proposed a mechanism of intermolecular recombination between homologous *trnA* loci with the simultaneous formation of chimeric pseudogenes to explain major inversions in rice. Whatever the causes, the distribution of plastome inversions can now be re-explored using full plastome sequences obtained by next-generation sequencing (NGS) technology.

While over 140 full grass plastomes have been assembled using traditional Sanger sequencing ([14–17]; and many others) and NGS [18–21], a complete plastome from other families within the graminid clade has yet to be sequenced. Here, the complete plastid chromosome of *Joinvillea ascendens* is sequenced and described. Evident in the arrangement of subgenomic sections are several large-scale molecular evolutionary events that are not present in grasses and the common cattail (*Typha latifolia*).

## Materials and Methods

### DNA extraction and plastome assembly

Silica-dried leaf material was provided by Dr. Lynn Clark, Iowa State University, Ames, IA under voucher (Clark & Attigala 1714). The tissue was homogenized in liquid nitrogen and DNA extraction was performed using the DNeasy plant minikit (Qiagen, Valencia, CA, USA) according to manufacturer instructions. Extractions were quantified using the Qubit fluorometric assay (Life Technologies, Grand Island, NY, USA) and diluted to 2.5 ng/ μL in 20 μL. Illumina libraries were prepared using the Nextera kit (Illumina, San Diego, California, USA) and sequenced single-end on a HiSeq 2000 platform at the Iowa State University DNA core facility (Ames, IA, USA).

Quality-trimmed reads were assembled into contiguous sequences (contigs) using Velvet v. 1.2.08 [22] iteratively by using a range of kmer sizes (19–85 by steps of six) and running Velvet using assembled contigs as input [23]. Since no reference plastome exists for any species of Joinvilleaceae, contigs could not be scaffolded by an automated method. Extensive *in silico* genome walking was needed, which follows a process of traversing a decision tree. This entailed manually following all possible branching decisions at the terminus of each contig to extend contigs into linear segments that ultimately formed a circular genetic map. A correct assembly would not lead to premature assembly of repeat regions, including the major inverted repeats, early termination, or incorrect insertion of repetitive sequences. Contigs were then extended by mapping reads and other assembled contigs in Geneious Pro version 7.1.8 (Biomatters Ltd., Auckland, New Zealand) until perfect overlap of at least 20 base pairs (bp) with other contigs or reads was obtained. This was repeated until the quadripartite plastome structure was completed.

The assembly was verified by mapping the quality-trimmed reads to the draft assembly in Geneious Pro. The consensus of that mapping was then compared to the sequence of the draft assembly to 1) detect inconsistencies in base composition 2) areas of discontinuity in the read mapping and 3) regions of low read depth. Read depth was found to exceed our minimum acceptable threshhold of 10 across the entire plastome assembly. The plastome was annotated by aligning plastid genes from two reference plastomes: *Anomochloa marantoidea* (NC014062), which is the sister lineage to all other grasses, and *Typha latifolia* (NC013823). *A*. *marantoidea* was used as the representative grass plastome because of its previous designation as a deeply diverging grass [24]. *T*. *latifolia* was used as a reference plastome for gene order (see below). The full plastome for *J*. *ascendens* was deposited in Genbank (KX035098). This was compared to the draft plastome of *J*. *plicata* [16], which comprises 20 contigs. The draft plastome had only 74.5% coverage of the complete plastome, and homologous portions were 95.2% identical as expected in congeneric species.

**Fig 1:**
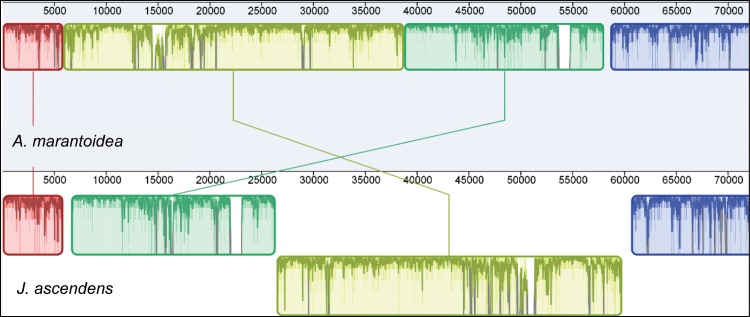
Diagram of the rearrangements present in the LSC region of the *J*. *ascendens* plastome compared to the grass *A*. *marantoidea*. This graphic was generated by the Mauve alignment package implemented in Geneious. Each pair of colinear regions are the same color and are positioned laterally as they exist in the plastome. The yellow region in *J*. *ascendens* that sits below the other three regions represents reverse-complementation.

BLASTN [25] was used to identify regions of significant sequence similarity between the *J*. *ascendens*, *A*. *marantoidea*, and *T*. *latifolia* plastomes. These regions were annotated and plastome rearrangements were first manually identified. The Mauve alignment algorithm [26] was then used with default settings to verify these rearrangements by identifying locally colinear blocks between these species in Geneious Pro (Fig 1). All rearrangement boundaries were inspected manually for a continuum of overlap and read coverage to identify potential assembly errors.

### Verification of rearrangement events

A computational approach was developed to verify inversions and to potentially uncover a more parsimonious series of evolutionary events. A custom Python script, which was named Detection of Inversion Modes through the Simulation of Unifying Mutations (DIMSUM), was produced to perform these confirmations. The script randomly inverted subsets of defined colinear regions (that were previously identified by Mauve) within a starting plastome 100 times until a predefined endpoint was reached. DIMSUM repeated this process a user-defined number times and reported all solutions that were reached. The Python script and documentation can be found at http://sourceforge.net/projects/grassplastome/.

## Results

### Sequencing the Joinvillea ascendens plastome

After trimming and filtering, the Illumina library generated 8,982,874 single-end reads of 25–100 bp (mean length: 92.4 bp). The completed *J*. *ascendens* plastome was 149,327 bp in length. The small and large-single copy regions were 12,907 and 85,526 bp respectively and the two inverted repeat regions were 25,447 bp each. The read depth of the plastome ranged from 19–147 with a mean depth of 77. The *J*. *ascendens* plastome had an AT composition of 60.4% and contained 117 protein coding sequences, 35 tRNA genes, and eight rRNA genes.

### Identification of major inversions

**Fig 2:**
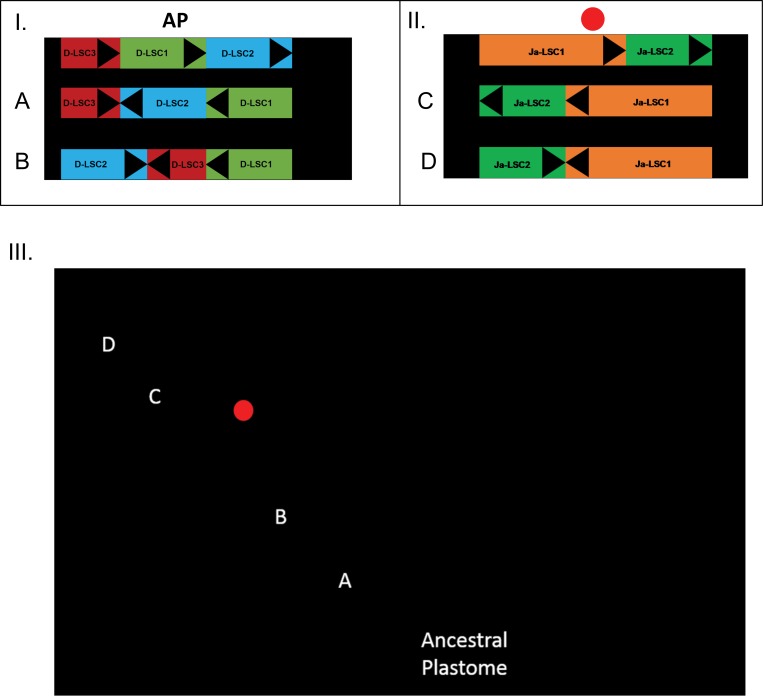
**I**–**II.** Diagram of the inversions that occurred within the large single-copy subregion of the plastome of the Joinvilleaceae-Poaceae lineage and *Joinvillea* lineage respectively. ‘AP’ denotes the ancestral plastome, which signifies the pre-inversion state (as observed in *Typha latifolia*) and the red circle signifies the ancestral plastome before the divergence between Joinvilleaceae and Poaceae. The arrows (A–D) represent large-scale (750–23,000 bases) inversion events. Triangular markers are placed on each colored region to demonstrate orientation. Subregions are not drawn to scale. **III.** A simplified cladogram representing the relationships between Joinvilleaceae, Poaceae, and Typhaceae. Arrows indicate the hypothesized relative position of each of the mutations (A–D) and one 300 base inversion exclusive to the grass lineage. The ‘ancestral plastome’ indication and red circle represent the positions of the hypothesized starting points from I and II. Branch lengths are not to scale.

*Anomochloa marantoidea* exhibited two large regions of rearrangement when compared to *T*. *latifolia*. One subregion within the LSC, between *trnfM*–*trnE* (~23 kbp; D-LSC1), was inverted in *A*. *marantoidea* and had laterally exchanged positions with the adjacent subregion between *psbD*–*trnfM* (~5 kbp; D-LSC2). A third region that contained *trnG* in *T*. *latifolia* (~750 bp; D-LSC3) bordered the upstream boundary of the inversion in *T*. *latifolia* and was rearranged to a reverse-complemented position between the two LSC rearrangements (Fig 2; I). The remaining portion of the LSC and IR regions exhibited colinearity. The SSC exhibited a similar pattern as the LSC with two subregions rearranged laterally with one region retaining its orientation (*ndhF*–*ndhH*, ~14 kbp; PoJo-SSC1) and the other reverse-complemented (*ndhH*–*ycf1*, ~3 kbp; PoJo-SSC2). This rearrangement was also present in all other grass subfamilies and in *J*. *ascendens*.

*Joinvillea ascendens* and *A*. *marantoidea* shared a colinear plastome except for one large region in the LSC domain between *rps16* and *psaI*. This region was divided into two homologous subregions of sizes ~33 kbp (*rps14*–*trnQ*-UUG; Ja-LSC1) and ~19.5 kbp (*psaB*–*rbcL*; Ja-LSC2). The two had rearranged laterally with the Ja-LSC1 subregion retaining its orientation and the Ja-LSC2 subregion exhibiting reverse-complementation (Fig 2; II).

### Unique plastome features

A functional copy of the *accD* gene, which encodes the beta-carboxyl transferase subunit of acetyl-CoA carboxylase [27] was not present in *J*. *ascendens*. In *J*. *ascendens*, *accD* was truncated to a pseudogene (*ψaccD*) approximately 66 amino acids (aa) upstream of the stop codon in its homolog in *T*. *latifolia*. The upstream portion of *ψaccD* was also truncated by an inversion break-point. *accD* was entirely absent from the *A*. *marantoidea* plastome.

The *ycf2* locus was also present in *T*. *latifolia* but absent in *A*. *marantoidea* except for two pseudogene fragments of 360 and 507 bp. The *ycf2* locus was mostly absent from the *J*. *ascendens* plastome except for two pseudogene fragments of 368 and 1641 bp. The *ycf1* locus is found intact in *T*. *latifolia*, but in *J*. *ascendens* is found as a pseudogene that is truncated by approximately 1,399 aa due to an internal stop codon created by a nonsense mutation (UAU → UAG). Approximately half of the downstream region of *ycf1* has been deleted in *J*. *ascendens*. The *ycf1* locus was entirely absent in *A*. *marantoidea*.

The *clpP* locus contained one intron in *J*. *ascendens* compared to two in *T*. *latifolia* and no introns in *A*. *marantoidea*. Additionally, a ~300 bp inversion between *A*. *marantoidea* and *T*. *latifolia* was observed in the *ycf3*–*trnS* intergenic spacer of the LSC.

## Discussion

### Large plastome rearrangements

Previous studies that documented monocot-specific plastid rearrangements used the plastome from the eudicot *Nicotiana tabacum* to represent the ancestral state [4, 11]. Here, we used the monocot *T*. *latifolia* due to its relatively closer phylogenetic proximity to the taxa of interest and consequent higher sequence similarity. Because our approach for inversion identification is based on homology inferred from sequence similarity, using *T*. *latifolia* as a reference point allows for a putatively more accurate inference of evolutionary events. A preliminary alignment of *T*. *latifolia* and *N*. *tabacum* indicated substantial conservation of gene order between the two taxa. Due to the similar synteny between *T*. *latifolia* and *N*. *tabacum* as well as phylogenetic divergence order within the angiosperms [1], we can infer that the gene order common to these two outgroups is ancestral compared to that of *J*. *ascendens*.

The largest documented inversion in the plastid LSC [4, 28] that united Restionaceae, Joinvilleaceae, and Poaceae was verified in this study as the event that inverted the D-LSC1 and D-LSC2 regions of *J*. *ascendens* (Fig 2; I-A). The second largest inversion, which united only Poaceae and Joinvilleaceae and which was documented in the same study, was also verified here as the event that returned D-LSC2 to the original orientation and inverted the adjacent D-LSC3 (Fig 2; I-B). The third and comparably smaller inversion (< 300 bp; unnamed), which is specific to the grass family, was also verified here.

The *J*. *ascendens* plastome exhibits a pattern of rearrangement consistent with two large inversions in the LSC region. The first inversion occurred in Ja-LSC1 and Ja-LSC2 (Fig 2; II-C) and the second occurred in Ja-LSC2, which returned it to the original orientation (Fig 2; II-D). Because no evidence of these two inversions is found in grass plastomes, these rearrangements are likely to have occurred after the divergence of the Joinvilleaceae and the Ecdeiocoleaceae + Poaceae lineage (Fig 2; III). Further sequencing of *Joinvillea* sp. plastomes would determine whether these rearrangements are synapomorphic to all members of the family or if they are specific to *J*. *ascendens* or another subclade of *Joinvillea*.

The rearrangement of the SSC region in Joinvilleaceae and Poaceae may suggest the occurrence of two inversions events. The first event would have inverted both PoJo-SSC1 and PoJo-SSC2 with a subsequent inversion of PoJo-SSC1 to the original orientation. These two events would have had to occur before the divergence of the common ancestor of Joinvilleaceae-Poaceae. The phylogenetic hypothesis in which Joinvilleaceae has a sister relationship to Poaceae + Ecdeiocoleaceae [1] would suggest that these inversion events should be evident in all members of Ecdeiocoleaceae. However, the exact placement of these inversions will remain obscured until sequences from additional monocot plastomes, such as *Flagellaria* sp., are determined. Alternatively, this pattern may be indicative of one inversion event, as the actual orientation of any SSC region at a given time cannot be determined with certainty due to its flanking IR regions. Typically, SSC regions are assembled to be alignable to previously sequenced plastomes, and the orientation of each single-copy region could be constantly in flux with homologous recombination of the IR regions [29]. Stein et al. [30] suggested that single-copy regions exist in equimolar populations of the two orientations.

When compared to *T*. *latifolia*, the *J*. *ascendens* plastome seems to have accumulated a fairly confounding series of inversions. However, when compared to *A*. *marantoidea* it becomes clear that the rearrangements in *J*. *ascendens* are a result of two large inversions that occurred in the Poaceae-Joinvilleaceae lineage followed by two large inversions that are specific to the Joinvilleaceae lineage. The two large inversions that were present in the Poaceae-Joinvilleaceae lineage are completely embedded in the ~33 kbp inversion specific to Joinvilleaceae, which explains why these inversions were not previously detected using PCR-based techniques with internal priming sites.

### Verification of rearrangement events

All series of inversions were shown to be the most parsimonious by running 1,000,000 replicates of DIMSUM. Although these events were initially found through visual inspection, DIMSUM was used to easily elucidate inversion events between sequences with more confounding steps and fewer collinear regions. Even within sequences with easily defined events, DIMSUM located the shortest series of inversions among a large number of sequences quickly and identified large-scale evolutionary patterns.

### Unique plastome features

No functional copies of *accD* were located in the remaining grass subfamilies, but remnants of the degraded copy were often present. Anomochlooideae, and all subfamilies within the “PACMAD” (Panicoideae, Aristidoideae, Chloridoideae, Micrairoideae, Arundinoideae and Danthonioideae) clade did not contain any remnants of *ψaccD*. The remaining grass subfamilies did contain *ψaccD* sequences that ranged from 59–451 bp. The structure of the *ψaccD* exhibits differential degradation between grasses and Joinvilleaceae, as seen in pseudogenes or other noncoding regions in independent evolutionary lineages of grasses [21, 31, 32]. An upstream region of *ψaccD* remained in *J*. *ascendens* and was deleted in all of the grasses and a downstream sequence remained in grasses, but was deleted in *J*. *ascendens*. Remaining pseudogene sequences also suggested two losses of ψ*accD* in Poaceae with one loss in the Anomochlooideae and a second loss in the PACMAD clade. A nearly identical region of *ψaccD* (~450 bp) is present in Pharoideae and Puelioideae, which suggests that this pattern of degradation is plesiomorphic due to the position of these two subfamilies as a basal grade. One study [33] reported that a larger remnant of *ψaccD* was found in the Chloridoideae, specifically within the genus *Eragrostis*. Previously published full plastomes from *E*. *tef* (KT168385) and *E*. *minor* (KT168384) exhibited no such sequence.

The structure of *clpP* in these taxa shows variation in intron number across taxa. The *T*. *latifolia* plastome has two introns in *clpP*, while *J*. *ascendens* and *A*. *marantoidea* have one and zero introns respectively. This may be indicative of a stepwise loss of introns in the grasses, with the first loss taking place in the Poaceae-Joinvilleaceae lineage followed by an independent loss in Poaceae. An alternative series of events, although less likely, could be the independent losses of one and two introns in Joinvilleaceae and Poaceae respectively.

### Conclusions

Major structural rearrangements are relatively rare in the plastomes of angiosperms, and have been most thoroughly explored in the graminid Poales. A hypothesized cause for large plastome inversions is recombination between dispersed tRNA loci with similar sequences [13]. However, not all of the major inversions observed in graminid Poales are flanked by tRNA genes or pseudogenes, so other mechanisms must be involved. Instances of reversals to the original sequence orientation (e.g. Fig 2; I, II) suggest that some type of persistent activating sequence feature may facilitate reversals. The role of selection has not been explored in these events, but preserving the proximity of promoters to their polycistronic plastid operons is a hypothetical selection pressure worth exploring. At present, the newly sequenced plastome can serve as a reference when assembling plastomes of other graminid clade Poales, such as *Joinvillea plicata* (Hook. f.), the type species of the genus, as well as species from Flagellariaceae and Ecdeiocolaeceae. Then the complete characterization of relatively rare structural rearrangements in the fully sequenced plastomes of graminid Poales would serve to better define branch points in the phylogeny of the group and more precisely delimit evolutionary lineages.
