# Correction: The First Complete Plastid Genome from Joinvilleaceae (*J*. *ascendens*; Poales) Shows Unique and Unpredicted Rearrangements

**DOI:** 10.1371/journal.pone.0166504

**Published:** 2016-11-08

**Authors:** William P. Wysocki, Sean V. Burke, Wesley D. Swingley, Melvin R. Duvall

Fig 2 does not display correctly in the published article. Please see the corrected Fig 2 here.

**Fig 2:**
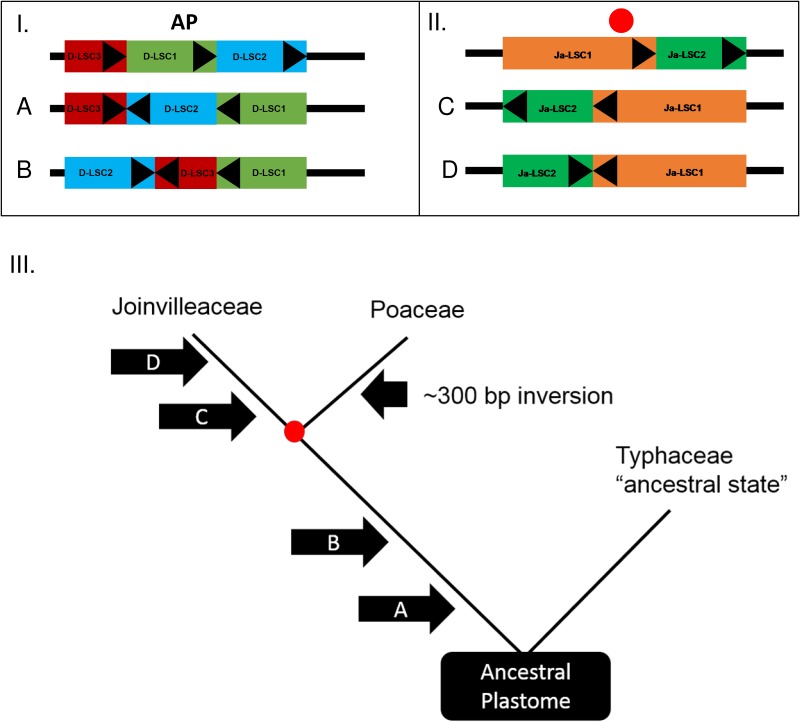
**I**–**II**. Diagram of the inversions that occurred within the large single-copy subregion of the plastome of the Joinvilleaceae-Poaceae lineage and *Joinvillea* lineage respectively. ‘AP’ denotes the ancestral plastome, which signifies the pre-inversion state (as observed in *Typha latifolia*) and the red circle signifies the ancestral plastome before the divergence between Joinvilleaceae and Poaceae. The arrows (A–D) represent large-scale (750–23,000 bases) inversion events. Triangular markers are placed on each colored region to demonstrate orientation. Subregions are not drawn to scale. **III**. A simplified cladogram representing the relationships between Joinvilleaceae, Poaceae, and Typhaceae. Arrows indicate the hypothesized relative position of each of the mutations (A–D) and one 300 base inversion exclusive to the grass lineage. The ‘ancestral plastome’ indication and red circle represent the positions of the hypothesized starting points from I and II. Branch lengths are not to scale.
